# Examining variation in the leaf mass per area of dominant species across two contrasting tropical gradients in light of community assembly

**DOI:** 10.1002/ece3.2281

**Published:** 2016-07-22

**Authors:** Margot Neyret, Lisa Patrick Bentley, Imma Oliveras, Beatriz S. Marimon, Ben Hur Marimon‐Junior, Edmar Almeida de Oliveira, Fábio Barbosa Passos, Rosa Castro Ccoscco, Josias dos Santos, Simone Matias Reis, Paulo S. Morandi, Gloria Rayme Paucar, Arturo Robles Cáceres, Yolvi Valdez Tejeira, Yovana Yllanes Choque, Norma Salinas, Alexander Shenkin, Gregory P. Asner, Sandra Díaz, Brian J. Enquist, Yadvinder Malhi

**Affiliations:** ^1^ École Normale Supérieure45, rue d'UlmF75005ParisFrance; ^2^ School of Geography and the EnvironmentEnvironmental Change InstituteUniversity of OxfordSouth Parks RoadOxfordOX1 3QYUK; ^3^ Department of BiologySonoma State University1801 East Cotati AvenueRohnert ParkCalifornia94928; ^4^ Wageningen University6708 PBWageningenThe Netherlands; ^5^ Universidade do Estado de Mato GrossoBR 158 km 650Nova XavantinaMato GrossoBrazil; ^6^ Universidad San Antonio Abad de CuscoAv. de la Cultura, Nro. 733CuscoPeru; ^7^ Sección QuímicaPontificia Universidad Católica del PerúAvenida Universitaria 1801San MiguelLima 32Peru; ^8^ Department of Global EcologyCarnegie Institution for Science260 Panama StreetStanfordCalifornia94305; ^9^ Instituto Multidisciplinario de Biología Vegetal (IMBIV)CONICET and FCEFyNUniversidad Nacional de CórdobaCasilla de Correo 4955000CórdobaArgentina; ^10^ Department of Ecology and Evolutionary BiologyUniversity of ArizonaTucsonArizona85721; ^11^ The Santa Fe Institute1399 Hyde Park RdSanta FeNew Mexico87501

**Keywords:** Community assembly, environmental filtering, interspecific variation, intraspecific variation, leaf mass per area, limiting similarity, tropical forests, *T*‐statistics

## Abstract

Understanding variation in key functional traits across gradients in high diversity systems and the ecology of community changes along gradients in these systems is crucial in light of conservation and climate change. We examined inter‐ and intraspecific variation in leaf mass per area (LMA) of sun and shade leaves along a 3330‐m elevation gradient in Peru, and in sun leaves across a forest–savanna vegetation gradient in Brazil. We also compared LMA variance ratios (*T*‐statistics metrics) to null models to explore internal (i.e., abiotic) and environmental filtering on community structure along the gradients. Community‐weighted LMA increased with decreasing forest cover in Brazil, likely due to increased light availability and water stress, and increased with elevation in Peru, consistent with the leaf economic spectrum strategy expected in colder, less productive environments. A very high species turnover was observed along both environmental gradients, and consequently, the first source of variation in LMA was species turnover. Variation in LMA at the genus or family levels was greater in Peru than in Brazil. Using dominant trees to examine possible filters on community assembly, we found that in Brazil, internal filtering was strongest in the forest, while environmental filtering was observed in the dry savanna. In Peru, internal filtering was observed along 80% of the gradient, perhaps due to variation in taxa or interspecific competition. Environmental filtering was observed at cloud zone edges and in lowlands, possibly due to water and nutrient availability, respectively. These results related to variation in LMA indicate that biodiversity in species rich tropical assemblages may be structured by differential niche‐based processes. In the future, specific mechanisms generating these patterns of variation in leaf functional traits across tropical environmental gradients should be explored.

## Introduction

Since the first reported field measurement of leaf mass per area (LMA) in 1917 (Hanson 1917), LMA has been a characteristic leaf trait used to understand the integration of light interception and plant growth (Poorter et al. 2009). Indeed, LMA has been found to be one of the most important traits that synthesizes the light capture dimension of the plant strategy spectrum (Grime et al. 1997; Westoby 1998; Wright et al. 2004; Díaz et al. 2016). Nutrient‐ or water‐depleted environments select for nutrient and water savings via long‐lived, thick, robust leaves with a high LMA (slow end of the leaf economic spectrum, LES), while nutrient‐rich environments favor more tender, faster‐growing (and often low‐LMA) leaves that are better able to outcompete potential neighbors (Wright et al. 2004; Poorter et al. 2009). As a result, LMA, or its reciprocal, specific leaf area (SLA) can be quite variable between species (Kazakou et al. 2014) and with environmental parameters (Poorter et al. 2009). Species‐level LMA has been shown to increase with latitude (Hulshof et al. 2013) and insolation (Ackerly 2004) and to decrease with temperature (Poorter et al. 2009) and rainfall (Warren et al. 2005). Importantly, intra‐individual variation in LMA has also been observed (e.g., 26% of total variation (Shipley 1995) and can be related to branch position in the canopy (Bruschi et al. 2003). Often, leaves located in shady areas of the canopy have lower LMA than leaves located in full sun from the same tree (Bruschi et al. 2003).

Since LMA of both sun and shade leaves captures fundamental trade‐offs in plant growth strategies, LMA is an important functional trait. Functional traits affect individual plant performance (i.e., growth, reproduction and survival), thus informing the structure and dynamics of vegetation (McGill et al. 2006; Ackerly and Cornwell 2007; Violle et al. 2007) as well as plant community responses to environmental variation (Lavorel and Garnier 2002) and competition (Kunstler et al. 2016). As a result, functional traits are often used by ecologists to disentangle community assembly mechanisms and ecological processes across contrasting environments (Diaz et al. 1998; Ackerly 2004; McGill et al. 2006; Anderson et al. 2011). Specifically, a quantitative comparison between intra‐ and interspecific variability in functional traits provides a novel framework to decipher community assembly patterns by distinguishing between internal and environmental filters (Jung et al. 2010; Paine et al. 2011; Aiba et al. 2013; Enquist et al. 2015). Functional traits and their associated metrics have been used to quantify multilevel (i.e., intraspecific, interspecific and community) assembly processes across latitudinal (Shepherd 1998; Swenson et al. 2012; Lamanna et al. 2014; Lawson and Weir 2014), climatic (Hulshof et al. 2013), elevation (McCain and Grytnes 2010; Körner 2007; Bryant et al. 2008), temporal (Enquist et al. 2015), and local vegetation gradients, such as forest–savanna transitions (Fernandes 2000; Ackerly et al. 2002).

Here, we measure intra‐individual, interspecific, and intraspecific variations in LMA across two contrasting environmental gradients. Specifically, we first investigate trait variation across (1) a forest–savanna transition in the Amazonia‐Cerrado contact area in Mato Grosso, Brazil, and (2) a 3300‐m elevation transect of undisturbed natural forest from the Andes to lowland Amazonia in southern Peru. We then explore patterns of intra‐ to interspecific variation in LMA (*T*‐statistics, Violle et al. 2012; Taudiere and Violle 2015) to evaluate broad hypotheses related to differing community assembly filters across these gradients. Despite the recognition that intraspecific variation is a key parameter in explaining a population's resilience to environmental changes and its ability to coexist and compete with its neighbors (Albert et al. 2010, 2011; Jung et al. 2010), few studies have proposed combining intra‐ and interspecific variability to understand diversity patterns across local and regional spatial scales (but see Paine et al. 2011; Hulshof et al. 2013; Le Bagousse‐Pinguet et al. 2014). Moreover, while studies have examined trait variation across elevation gradients (Niinemets 2001; Anderson et al. 2011; Hulshof et al. 2013; Asner et al. 2014a) and forest–savanna transitions (Hoffmann et al. 2005; Carlucci et al. 2015), and some have focused on trait variation in relation to biotic and abiotic drivers (Poorter et al. 2009; Hulshof et al. 2013; Kichenin et al. 2013; Read et al. 2014), our study is the first to use trait variance ratios via *T*‐statistics to explore community assembly over a continuous gradient and across multiple sites, each within the same biogeographic region in the tropics.

A suite of functional trait metrics exist (Aiba et al. 2013), but *T*‐statistics are particularly useful for our type of study as they partition functional trait variation among spatial and taxonomic levels in a straightforward way (Violle et al. 2012). Furthermore, in conjunction with *T*‐statistics, a suite of flexible null models can be used to detect nonrandom patterns of functional traits and to draw inferences about hypotheses of community assembly related to environmental filtering and species interactions (Taudiere and Violle 2015). While *T*‐statistics have been recently proposed as a novel method to investigate community assembly, we recognize that *T*‐statistics must be handled with care in regard to interpretations for our study system. First, while community ecologists traditionally distinguish between biotic and abiotic filtering, *T*‐statistics only indicate the presence or absence of processes that are internal and external to the community. In addition, *T*‐statistics are not independent from each other, as they are different combinations of a few variance components (VCs). Yet, with a knowledge of the environmental context of the study and a careful examination of individual VCs, data interpretation using *T*‐statistics is useful. In this regard, we used *T*‐statistics to investigate whether observed LMA patterns could be interpreted to help inform and derive hypotheses related to community assembly.

Based on existing literature, we hypothesize that LMA is greater in less productive environments and thus increases with elevation (e.g., Hulshof et al. 2013) and in savanna compared to forest sites. Across broadscale climate gradients, especially from the tropics to the temperate zone (Fischer 1960), environmental filtering is hypothesized to be a major driver of the structure and functioning of ecosystems (von Humboldt and Bonpland 1807; Schimper and Andreas 1908; Solbrig 1994; Kleidon and Mooney 2000; McGill et al. 2006; Malhi et al. 2010; Kerkhoff et al. 2014; Enquist et al. 2015). Thus, considering the strong environmental variation among tropical sites, we hypothesize that environmental filters play an important role community assembly – from cold, high‐elevation sites in Peru to hot and seasonally dry savanna sites in Brazil. Since species richness is greater within the Brazilian forest site and at the Peruvian low‐elevation sites, and thus more competitors are established in these areas, we expect internal filters to nonrandomly affect community assembly in these plots as a result of a reduction in local intraspecific variation (MacArthur and Levins 1967).

## Materials and Methods

### Study sites

The forest–savanna gradient (Table 1) was located in the vicinity of Nova Xavantina, Mato Grosso, Brazil. The region is characterized by two well‐defined seasons: hot and wet from October to March, and cool and dry from April to September (Marimon et al. 2014). The area sits at the ecotone between Amazonia and Cerrado biomes and has a rapid transition from an Amazonian‐type seasonal forest to an open‐canopy, sensu stricto *cerrado* (South American woody savanna); local abrupt transitions in vegetation type are mediated by differences in soil physical and chemical properties (Marimon Junior and Haridasan 2005). Three of the four 1‐hectare plots were located in Parque Municipal do Bacaba (14°41′S 52°20′W) in Nova Xavantina, Mato Grosso, and represented three distinct vegetation types of progressively decreasing woody biomass and stature (*cerradão*,* cerrado tipico*, and *cerrado rupestre*, see Marimon Junior and Haridasan 2005). The fourth site was located in a semi‐deciduous forest, located 25 km away (SE) from Nova Xavantina in the reserve of Fazenda Vera Cruz (14°50′S and 52°08′W) (Marimon Junior and Haridasan 2005; Marimon et al. 2014).

**Table 1:** Environmental and sampling data for the 10 plots in Peru (Malhi et al. 2016 and unpublished data) and the four plots in Brazil (Marimon et al. 2014 and unpublished data)

Country	Site code	Vegetation type	Latitude	Longitude	Elevation^a^ (m)	Mean annual air temp (°C)	Precipitation (mm·year^−1^)	Soil type (WSRD)	Number of species (sampled / total)	Number of trees (sampled / total)
Peru	TAM‐06	Lowland forest	−12.8385	−69.2960	215	24.4	1900	Alisol	17 / 175	47 / 646
	TAM‐05	Lowland forest	−12.8309	−69.2705	223	24.4	1900	Cambisol	25 / 157	71 / 530
	PAN‐02	Submontane forest	−12.6495	−71.2626	595	23.5^b^	2366^b^	Plinthosol	14 / 159	39 / 582
	PAN‐03	Submontane forest	−12.6383	−71.2744	859	21.9^b^	2835^b^	Alisol	15 / 153	33 / 682
	SPD‐02	Lower cloud forest	−13.0491	−71.5365	1494	18.8	5302	Cambisol	26 / 143	75 / 794
	SPD‐01	Lower cloud forest	−13.0475	−71.5423	1713	17.4	5302	Cambisol	29 / 153	73 / 1127
	TRU‐04	Upper cloud forest	−13.1055	−71.5893	2719	13.5	2318	Umbrisol	17 / 52	76 / 940
	ESP‐01	Upper cloud forest	−13.1751	−71.5948	2868	13.1	1560	Umbrisol	13 / 53	61 / 842
	WAY‐01	Upper cloud forest	−13.1908	−71.5874	3045	11.8	1560	Umbrisol	11 / 51	42 / 1165
	ACJ‐01	Tree line forest	−13.1469	−71.6323	3537	9.0	1980	Cambisol	11 / 28	45 / 856
Brazil	VCR‐02	Semi‐deciduous seasonal forest	−14.830	−52.130	294	25	1400	Plinthosol	12 / 73	39 / 471
	NXV‐02	Cerradão (transitional forest)	−14.702	−52.352	314	25	1400	Ferrosol	17 / 107	49 / 1671
	NXV‐01	Cerrado sensu stricto (savanna with small trees, shrubs, and grass understory)	−14.708	−52.353	325	25	1400	Ferrosol	29 / 108	87 / 2992
	CRP‐01	Cerrado rupestre (shrub‐ and grass‐dominated savanna)	−14.713	−52.352	372	25	1400	Lithic leptosol	20 / 80	63 / 1572

a Derived from high‐resolution airborne Light Detection and Ranging (LiDAR) data (see Asner et al. 2014aa for methodology).

b Derived from observations between 6 February 2013 and 7 January 2014.

In Peru, we sampled ten 1‐ha plots (Table 1) along elevation gradients in the departments of Cusco and Madre de Dios in SE Peru. Six of the plots are montane plots in the Kosñipata Valley, spanning an elevation range 1500–3500 m (Malhi et al. 2010), two are submontane plots located in the Pantiacolla front range of the Andes (range 600–900 m) and two plots are found in the Amazon lowlands in Tambopata National Park (elevation range 200–225 m). All plots are operated by the Andes Biodiversity Ecosystems Research Group (ABERG, http://www.andesconservation.org) and are part of the RAINFOR (www.rainfor.org) and Global Ecosystems Monitoring Network (GEM; http://gem.tropicalforests.ox.ac.uk) networks. Plots are located in areas that have relatively homogeneous soil substrates and stand structure, and which have minimal evidence of human disturbance (Girardin et al. 2014b). The montane plots were established between 2003 and 2013 and the two lowland plots were established in 1983. During plot establishment, all stems ≥10 cm diameter at breast height were tagged and identified to species level and, in recent years, plots have been measured at monthly intervals for carbon allocation and cycling following standard the GEM Network protocol (Marthews et al. 2014). As such, net primary productivity estimates (Girardin et al. 2010) and comprehensive descriptions of the carbon cycle exist for many of these plots (Girardin et al. 2014a; Huaraca Huasco et al. 2014; Malhi et al. 2014, 2016). From February 2013 to January 2014, mean annual air temperature varied from 9 to 24.4°C along the gradient and precipitation ranged from 1560 to 5302 mm·year^−1^ across all sites along the gradient (Table 1). Precipitation peaks strongly at mid‐elevations (around 1500‐m elevation).

### LMA measurements

Leaf mass per area was measured from April to November 2013 in Peru and from March to May 2014 in Brazil. In both countries, based on the most recently available census and diameter data, a sampling protocol was adopted wherein species were sampled that maximally contributed to plot basal area (a proxy for plot biomass or crown area). We aimed to sample the minimum number of species that contributed to 80% of basal area (Pérez‐Harguindeguy et al. 2013), although in the diverse lowland forest plots in Peru, we only sampled species comprising 60–70% of plot basal area. In Peru, within each species, 3–5 individual trees were chosen for sampling (five trees in upland sites and three trees in lowland sites). If three trees were not available in the chosen plot, we sampled additional individuals of the same species from an area immediately surrounding the plot. In Brazil, three trees from each species were sampled from within each plot. Using single‐rope tree climbing techniques, we sampled one fully, sunlit canopy branch, at least 1 cm diameter, from each tree. In Peru, we also sampled one branch from the inner, shady canopy if available; this was not the case in Brazil where very open canopy made the distinction between sun and shade branches impossible. In Peru, we measured five leaves from simple‐leaved species, or five individual leaflets from compound‐leaved species (both referred to as “leaf” below) from each branch (sun and shade) for trait measurements. In Brazil, three leaves were measured from each sun branch. Leaves were removed from the branch after branch cutting and immediately measured for LMA in a field laboratory. If transport back to the field laboratory from the field plot was required, leaves were placed in coolers until measurements could proceed. In the case of compound leaves, the entire compound leaf was also collected for whole‐leaf area calculations. Branches and leaves were chosen with minimal damage from herbivory.

Lamina and petiole (and rachis for compound leaves) were cut and scanned separately on a flatbed scanner (Canon Lide 110^®^) to measure leaf fresh area as described below. When leaves were larger than the scanner bed, they were cut into smaller pieces that were scanned separately. Once fresh area was measured, leaves were oven‐dried at 72°C until constant weight was reached (around 72 h), and their dry mass then was measured.

Leaf area images were processed using NIH ImageJ (Schneider et al. 2012) from within a custom MATLAB script (version R2013a). Specifically, each image was first binarized using an automatic threshold, visually checked, and the threshold then set manually to fit the leaf shape as accurately as possible. When the leaf was damaged (i.e., due to herbivory), the holes were not filled.

### Leaf area index measurements

In Brazil, leaf area index (LAI) measurements were taken as a proxy for light availability. LAI values and gap fraction per plot were calculated from 25 hemispherical pictures per plot taken in the center of each 20 × 20 m subplot with a Nikon 5100 camera and a Nikon FishEye Lens of 8 mm (Nikon U.K. Ltd, Surrey, U.K). Images were processed using the software Hemisfer (registered version, Schleppi et al. 2007; Thimonier et al. 2010). LAI measurements were not available for Peruvian sites at the time of these analyses.

### Statistical analyses

#### Means and variances

Leaf mass per area (dry mass [g]/fresh area [m^2^]) was calculated on a whole‐leaf basis, including both laminas and petioles. In cases where dry petioles had a mass of zero (due to a balance with limited precision), the dry masses were corrected by the site‐level ratio of fresh mass/dry mass of the petiole. For the statistical analyses, leaf areas from images with folded leaves were not included. Leaves that caused the tree to have variances >2000 g^2^·m^−4^ for LMA were also not included (i.e., single leaves from groups of five leaves were removed as outliers) when investigation of raw data strongly suggested measurement error.

Leaf mass per area was calculated separately for each leaf before calculating the mean LMA per individual tree. At the species and plot levels, LMA was calculated from individual tree (and not leaf) values. Significance threshold was set at *α = *0.05. In order to compare sites and to account for species abundance, community‐weighted means and variances (CWMs; Violle et al. 2007; Hulshof et al. 2013) were used:

$$\text{CWM}k=\Sigma_{i}\mu_{i}f_{i},$$

where *k *= plot, *i *= species, and *μ*_*i*_ and *f*_*i*_ are the mean trait value and relative abundance of the species *i* (proportion of basal area). Due to sampling constraints, the total sampled abundances differed among plots; that is, the sum of all *f*_*i*_ varied, making the comparisons between plots difficult. To correct this sampling bias, the final CWM for each plot was normalized by the sum of total abundances.

#### Variance decomposition

In order to avoid potential scaling effects between mean and variance, we used the log of LMA for the following variance decomposition analyses. We utilize Violle et al.'s (2012) three phenotypic variance ratios, termed the *T*‐statistics (to echo the *F*‐statistics in population genetics, “*T*” referring to traits), to account for intraspecific variation, relative to interspecific variation. Building on MacArthur and Levins' (1967) findings that the relative importance of intra‐ and interspecific phenotypic variation is a key parameter of species coexistence, these variance ratios test for internal and environmental filtering of a given community at different spatial and organizational scales (e.g., individual, species, whole community) (Violle et al. 2012). For each community (plot), we calculated three *T*‐statistics values (Violle et al. 2012) using the *cati* R package (function *Tstats* in cati). If A and B are two different levels of inclusiveness (i: individual, p: population, c: community, r: region) and $\sigma_{AB}^{2}$ is the variation of *A* within *B*, then:

- *T_*ip.ic = $\sigma_{\mathrm{IP}}^{2}/\sigma_{\mathrm{IC}}^{2}$, or the ratio of within‐population variance to total within‐community variance, measures niche packing among the species of the community and reflects internal filtering affecting individuals.
- *T_*ic.ir = $\sigma_{\mathrm{IC}}^{2}/\sigma_{\mathrm{IR}}^{2}$, or the ratio of community‐wide variance to total variance in the regional pool, measures external filtering strength when accounting for individuals.
- *T_*pc.pr = $\sigma_{\mathrm{PC}}^{2}/\sigma_{\mathrm{PR}}^{2}$, or the ratio of community‐wide variance to total variance in the regional pool, measures external filtering strength when only accounting for species (no intraspecific variation).

To determine the significance of the *T*‐statistics, results from Peru and Brazil were compared to null models generated from within the *cati* package using standardized effect sizes (SESs; function *ses* in cati). Details on null models can be found in Table S1 in Supporting Information. We defined the regional pool separately for Brazilian and Peruvian sites so that all of the trait values observed across each whole transect were a different regional pool (Taudiere and Violle 2015). Since the definition of a species pool can impact our conclusions (Lessard et al. 2012), we repeated our analyses with different regional pool sizes (i.e., not including all of the sites along the gradients within countries). Nonetheless, our results were not sensitive to regional pool size (data not shown).

As proposed by Messier et al. (2010), we used a restricted maximum‐likelihood method to decompose the variance of LMA on different levels: leaf, tree, species, site, and country. The *lme* and *varcomp* functions (R packages nlme and ape) were used to successively perform a nested ANOVA with random effects and apply a VC analysis. The R code used to calculate the variance partitioning on the whole dataset is:

$$\begin{array}{cc} \text{VC} & =\text{varcomp}(\text{lme}(\text{LMA}∼1,\;\text{random}\\ & =∼1|\text{Site}/\text{Family}/\text{Genus}/\text{Species}/\text{Tree},\;\text{data}\\ & =d,\;\text{na.action}=\text{na.omit})). \end{array}$$

Next, to account for species turnover along the gradients of dissimilarity, we calculated the Sorensen index (R package betapart, function *beta.pair)*. The obtained distance matrix was then compared with a distance matrix of the main quantifiable environmental parameters (elevation in Peru and LAI in Brazil). Significance was calculated using a Mantel test (R package vegan). The proportion of variance due to species turnover was also calculated following Leps et al. (2011) approach, with the *decompCTRE* function of the package *cati*. All above analyses were conducted using R version 3.0.2 and R scripts are included as online supporting information.

## Results

### Variation in LMA across environmental gradients

Across the Brazilian vegetation gradient at the species level, LMA varied from 20.7 (± standard error 6.2 g·m^−2^, *Faramea Torquata* PAN‐03) to 263.2 ± 62.2 g·m^−2^ (*Emmotum nitens*, NXV‐01). Site‐level LMA (i.e., mean LMA of all measured trees) in the forest (VCR‐02) and transition (*cerradão*; NXV‐02) plots was not significantly different, with respective means of 111.0 ± 28.6 and 116.8 ± 40.6 g·m^−2^. Plot‐level LMA in the savanna (NXV‐01) and dry savanna (CRP‐01) plots differed significantly from each other (*P *=* *0.006) and from the forest sites (*P *<* *2.10^−6^), with respective means of 147.5 ± 34.8 and 130.1 ± 34.6 g·m^−2^ (Fig. S1).

In Peru, tree‐level LMA from sun leaves varied from 19.9 ± 4.9 g·m^−2^ (*Senefeldera inclinata* in PAN‐03) to 392.2 ± 29.2 g·m^−2^ (*Persea ferruginea* in ACJ‐01). For shade leaves, tree‐level LMA varied from 17.6 ± 2.0 g·m^−2^ (*Senefeldera inclinata* in PAN‐03) to 248.9 ± 30.7 g·m^−2^ (Fig. S1). In both cases, LMA increased significantly with elevation, although elevation explained only part of the variation in LMA (sun *r* ^2^ = 0.16, shade *r* ^2^ = 0.21, *P *<* *0.001). Mean LMA was higher in sun leaves (141.9 ± 50.8 g·m^−2^) than in shade leaves (112.1 ± 41.4 g·m^−2^, *P* < 0.05), even when correcting for site variation. The magnitude of variation between sun and shade leaves did not vary between sites. Observed variation in LMA across Brazil and Peru represents a significant fraction of the variation in global LMA values (14–1500 g·m^−2^) reported by Wright et al. (2004).

**Figure 1:**
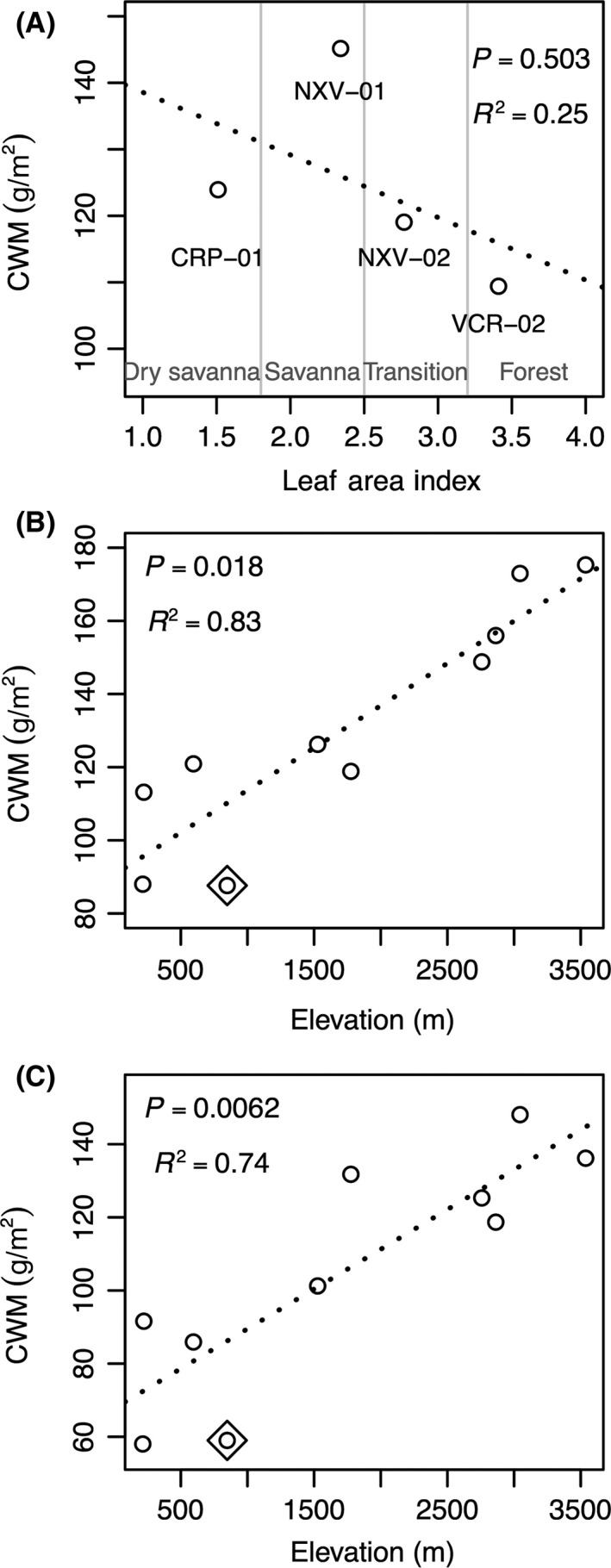
Community‐weighted mean (CWM) of leaf mass per area (LMA, g·m^−2^) along the (A) forest–savanna vegetation gradient in Brazil and 3300‐m elevation gradient from the Andes to the Amazon in Peru for (B) sun and (C) shade leaves, respectively. Each dot represents one plot (community); the boxed dot in (B) and (C) represents PAN‐03, a plot with particularly low LMA. *P* and *r* ^2^ values are provided for the relationship between (A) CWM and leaf area index in Brazil, and (B) and (C) CWM and elevation in Peru.

Community‐weighted LMA followed similar patterns as unweighted LMA. In Brazil, CWM LMA was lower in the forest than both savanna sites, with the highest CWM in the savanna (NXV‐01; Fig. 1A). No significant linear variation was found between CWM LMA and LAI measurements. In Peru, CWM LMA also increased significantly with elevation for both shade and sun leaves. One site (PAN‐03) appeared to have a significantly lower CWM LMA than the other sites. With this site removed from the analysis, elevation explained an important part of the variation in the CWM (sun leaves: *r* ^2^ = 0.77, *P *<* *0.001; Fig. 1B; shade leaves: *r* ^2^ = 0.68, *P* < 0.001, Fig. 1C). The trend was significant even when this site was included (*P* < 0.05, *r* ^2^ = 0.72 [sun leaves] or 0.46 [shade leaves]).

### Variance partitioning and species turnover

A very high species turnover was observed along both environmental gradients, with a Sorensen dissimilarity index between pairs ranging from 33 to 95% in Brazil and 63 and 100% in Peru (Fig. S2 in Supporting Information). Consequently, in both Peru and Brazil, the first source of variation in LMA was species turnover (65% in Brazil, 89% for sun leaves and 82% for shade leaves in Peru, Fig. 4). When variance decomposition was calculated with nested levels, inter‐ and intraspecific variation explained the majority of variance in LMA (Fig. S3 in Supporting Information). Variation at the genus or family levels was greater in Peru than in Brazil (Fig. S3).

### Exploration of internal and environmental filters

**Figure 2:**
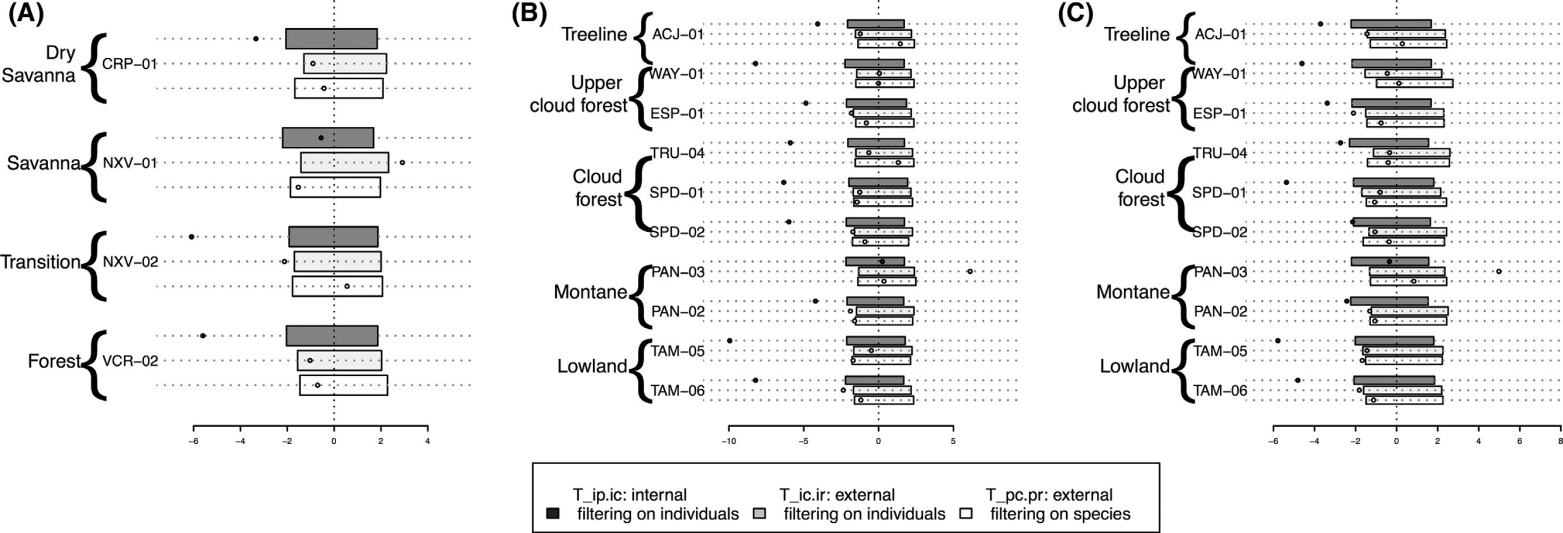
Standardized effect size (SES) of *T*‐statistics (see figure legend) of LMA along the (A) vegetation and elevation gradients for (B) sun and (C) shade leaves. Here, for each community or plot, we report three observed and null *T*‐statistics: (1) the ratio of the trait variance of individuals in species groups to the trait variance of individuals in the local community (*T_*ip.ic, dark shaded box); (2) the ratio of trait variance of individuals in the local community to the trait variance of the individual in the whole regional pool (*T_*ic.ir, striped dark gray box); and (3) the ratio of the species trait variance in the local community to the species trait variance in the regional pool (*T_*pc.pr, solid light gray box). Each filled dot represents the mean SES value for one community (plot) compared to the null model expectation. Boxes indicate the confidence interval of the null model for each *T*‐statistic. The SES are significantly different from the null distribution if *not* embedded within the box. The lower the SES value compared to the null model, the stronger the filter.

In Brazil, in all plots except the savanna plot, the *T*‐statistic *T_*ip.ic (measuring the relative strength of internal filtering on individuals) was lower than expected by the null model. This suggests that internal filters (e.g., competition) between individuals within populations (Fig. 2A) may impact community assembly. Moreover, *T_*ip.ic had a lower SES (i.e., lower trait variance) in the forest and forest–savanna transition plots than in the savannas. This was due to a lower intraspecific variation in forests than in savannas (Table S3), which might indicate a stronger internal filter on individuals. In the savanna plot (NXV‐01), individual variation was high at both species and community levels. This caused the second *T*‐statistic *T_*ic.ir (measuring the relative strength of external filtering on individuals) to be greater than expected in this plot due to a very high variation at the individual level compared to the regional level. This could be due either to very low external filtering in this plot or to the presence of transitional and local species, resulting in high trait variation. Significant differences between values of the third *T*‐statistic *T_*pc.pr (measuring the relative strength of external filtering on species) and the null model were not observed at any plot in Brazil.

In Peru, patterns were extremely similar for sun and shade leaves. For sun leaves, the first *T*‐statistic *T*_ip.ic was significantly lower than expected from the null model in all plots except PAN‐03 (which has an exceptionally high intraspecific variance, cf. Table S3, Fig. 1B). The second *T*‐statistic, *T_*ic.ir, was slightly lower than the null model in 4 plots: ESP‐01, SPD‐02, PAN‐02, and TAM‐06. It was higher than expected from the null model in PAN‐03 due, again, to high individual variance. Similar to the Brazilian plots, external filtering on species was not observed since the third *T*‐statistic, *T_*pc.pr, was not significantly different from the null model. There was no clear pattern of variation in SES with elevation for any *T*‐statistic along this gradient.

**Figure 3:**
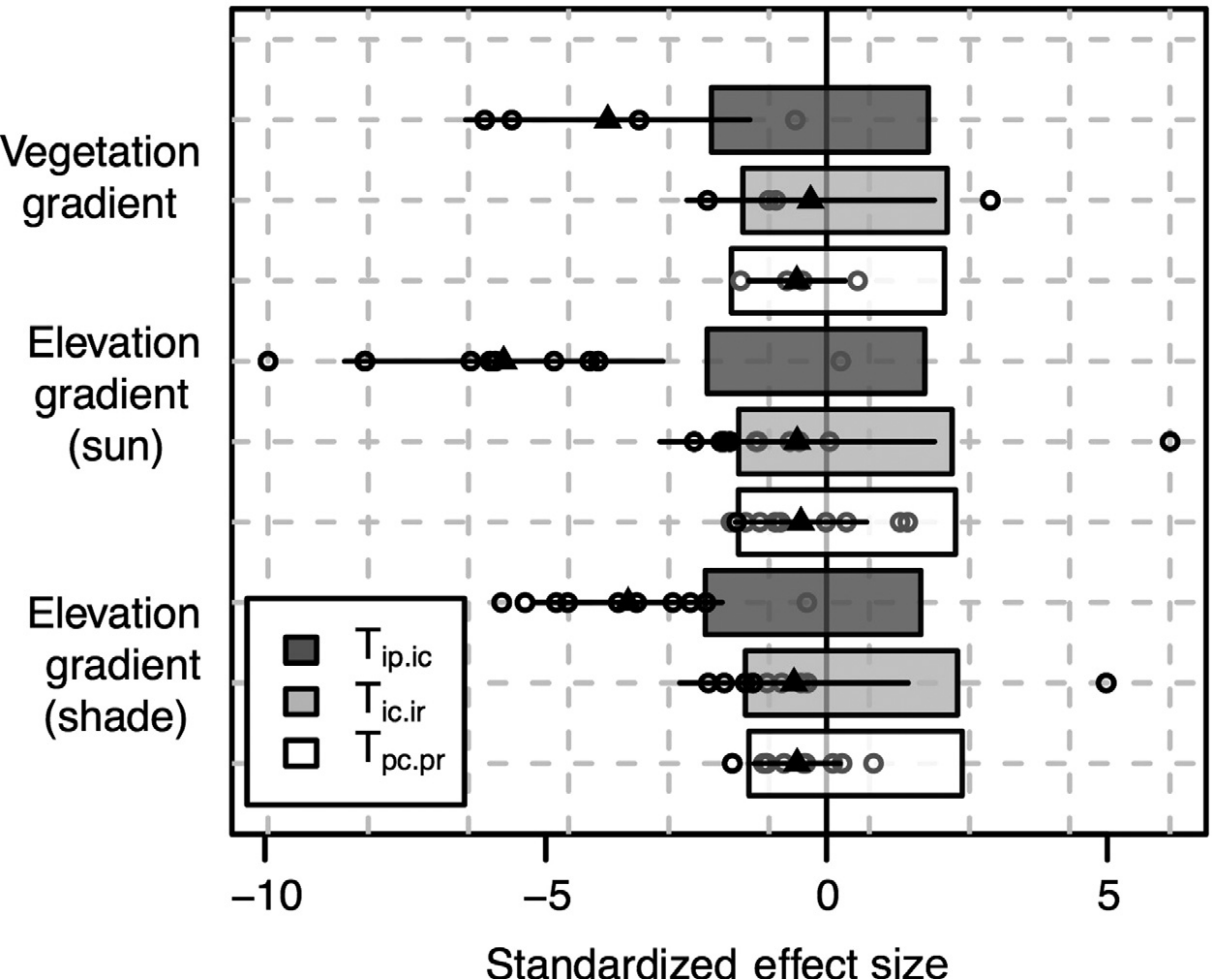
Standardized effect size (SES) of *T*‐statistics of LMA along the entire elevation gradient in Peru for sun and shade leaves and vegetation gradient in Brazil. Each dot represents the SES value for one community (plot) along the gradient. The triangles and the segments represent, respectively, the mean and the standard deviation of the SES values for a given *T*‐statistic (i.e., mean and standard deviation of community values). Boxes delimitate the confidence interval of the null model for the whole gradient; thus, for a given *T*‐statistic, the mean of the SES (crossed circle) is significantly different from the null distribution if it is not embedded within the box. *T_*ip.ic: ratio of within‐population variance to total within‐community variance, *T_*ic.ir: community‐wide variance relative to the total variance in the regional pool, and *T*_pc.pr: intercommunity variance relative to the total variance in the regional pool.

In general, the mean difference in SES for *T_*ip.ic from the null model in Peruvian sun leaves seemed greater than the mean difference in SES in Brazil, although this difference was not significant (Fig. 3). The SES in sun leaves were significantly lower than shade leaves (*P* = 0.018). The mean SES for *T_*ic.ir and *T_*pc.pr were never different from null model.

## Discussion

### Variation in LMA and community‐weighted LMA

#### Brazil

In Brazil, community‐level LMA was greater in savanna sites compared to the forest site, consistent with previous studies (Niinemets 2001; Ackerly et al. 2002; Hoffmann et al. 2005). This shift supports the hypothesis that dry, highly insolated, infertile environments promote long‐term investments and high‐LMA leaves (Wright et al. 2004). Indeed, greater LMA in savannas compared to forested areas has been attributed to rainfall (Wright and Westoby 2002; Warren et al. 2005; Baruch 2011), light (Ackerly et al. 2002), soil fertility (Baruch 2011), and phylogenetic differences (Hoffmann and Franco 2003; Hoffmann et al. 2005). As rainfall is equivalent across plots, and Marimon Junior and Haridasan (2005) showed that the savanna and forest–savanna transition sites (NXV‐01 and NXV‐02) had similar soil fertilities, soil water availability (as it relates to soil texture and not direct precipitation) may explain observed LMA patterns. In support of this hypothesis, Marimon Junior and Haridasan (2005) showed that a higher clay content in NXV‐02 (the site with the highest LMA) adversely affected water availability, water balance, and water stress response of woody plants – all of which could directly impact leaf morphology.

In addition to water stress, environments with low light availability have been found to select for low‐LMA leaves (Ellsworth and Reich 1992; Niinemets 2001). However, LMA was higher in the savanna (NXV‐01) than in the dry savanna site (CRP‐01), which had less trees, more shrubs, and grasses, and a more rocky terrain (Maracahipes et al. 2011, B.S. Marimon, pers. comm). Thus, greater LMA in the dry savanna might instead be due to a difference in deciduousness between plots, as more species in the savanna (CRP‐01) lose leaves in the dry season (Table S2 in Supporting Information) and a smaller investment in deciduous leaves could result in less dense leaves.

Lastly, low LMA in the forest plot compared to the savanna, dry savanna, and transition plots could relate to species composition (Hoffmann and Franco 2003; Hoffmann et al. 2005). Species turnover was relatively low in all plots except the forest plot (Fig. S2a). It is thus likely that greater LMA in plots other than the forest plot is due the presence of high‐LMA species, consistent with the presence of transitional species in the savanna and transition plots. Carlucci et al. (2015) also found that nearly half of the variation in the community‐weighted SLA was explained by the turnover of species between plots in the Pampa biome in southern Brazil.

#### Peru

In Peru, patterns observed for LMA and community‐weighted LMA with elevation were consistent with other studies in both temperate and tropical regions (Hultine and Marshall 2000; Asner et al. 2011, 2014a; Hulshof et al. 2013; Asner and Martin 2016). Increasing LMA with increasing elevation (Fig. 1) is consistent with assumptions of the leaf economic spectrum (Wright et al. 2004) as higher LMA corresponds to a slow‐end strategy (long‐term investment and nutrient retention) expected in less productive environments: lower temperature, increased UV exposure, and changes in precipitation and their combinations could all affect LMA at high elevation.

Unweighted mean LMA was also higher in sun leaves than shade leaves. This can also be related to the leaf economic spectrum, with sun leaves being subject to higher UV exposure. Elevation explained a more important part of community‐weighted LMA variation in sun (53% for all sites, 83% without PAN‐03) than shade leaves (72% for all sites and 43% without PAN‐03). This supports the idea that light availability, which is the main parameter varying between sun and shade leaves, might be one of the main drivers of LMA variation with elevation.

Interestingly, the contribution of elevation in explaining the variance in LMA was weaker for unweighted LMA compared to community‐weighted LMA (e.g., 15% vs. 72%, respectively, for sun leaves, all sites included; and 21% vs. 46% for shade leaves). Differences in weighted and simple averages of traits have been found in other systems and attributed to environmental filters acting more strongly on dominant than on rare species (Cingolani et al. 2007). Thus, the stronger relationship of CWM LMA with elevation compared to the unweighted mean could result from more individuals at high altitudes with higher LMA. This could then cause CWM values to increase whether either each of the species present in more or less equal abundance at high altitude has high LMA, or whether there are different species with different LMA values, but those with high LMA are present in a higher abundance. Alternatively, our observed results are possibly due to sampling methodology. Only five leaves from a single branch of the tree were used to calculate both unweighted and CWM LMA. In a related study along this gradient in Peru where a bulk leaf sample was collected from multiple branches and leaves within a single tree, unweighted LMA and CWM LMA are similar within plots (Asner and Martin 2016). Thus, our limited sample might be less robust to intraspecific variation.

**Figure 4:**
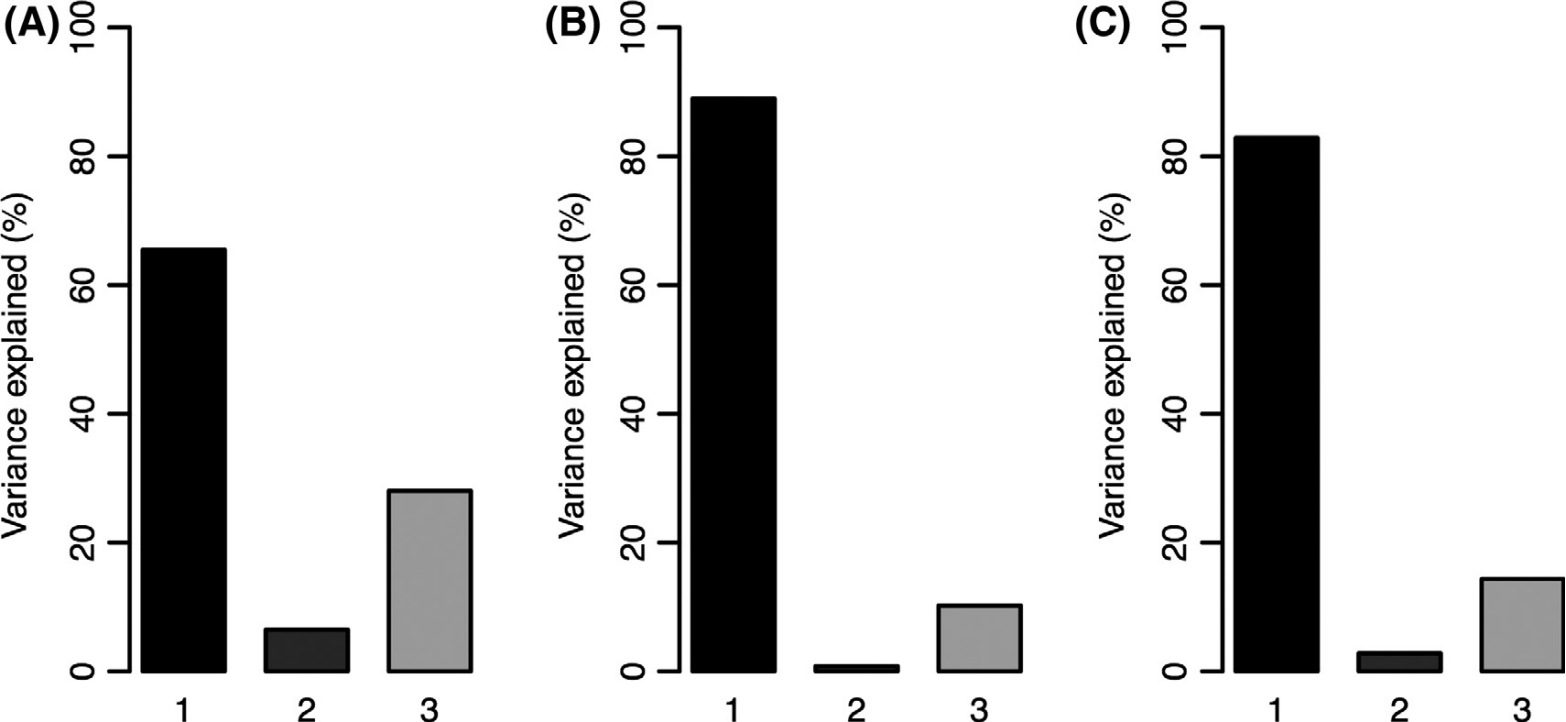
Decomposition of variation for leaf mass area (LMA) in (A) Brazil and Peru in (B) sun and (C) shade leaves. Species turnover (1), intraspecific variation (2), and their covariance (3) were included using the Leps et al. (2011) approach.

Regardless of differences in variation between unweighted and CWM LMA, strong turnover, rather than a direct effect of environmental filters, could be explaining changes in LMA with altitude. Indeed, variation in LMA was more likely due to variation in species abundance rather than to specific trait variation (Pescador et al. 2015), as species turnover was high along the gradient in Peru (Fig. 4). The decomposition of variance for LMA in Peru supports a strong relationship between variation in LMA and phylogenetic variation (Scheepens et al. 2010; Fajardo and Piper 2011) since variation in family, genus, and species alone explained over 75% of the variation in LMA for sun leaves and 60% for shade leaves (Fig. S3). Other trait‐based studies along this gradient in Peru corroborate the importance of site differences in species and genera in explaining trait variation (Asner et al. 2014b). In fact, phylogeny was found to explain about 52% of variation in LMA among all sites along the gradient, and the explanatory power of phylogeny was often higher within sites (78% at TAM sites; Asner and Martin 2011).

### Internal and environmental filtering in Brazil and Peru

Using *T*‐statistics applied to LMA variation of the dominant species, we were able to determine the presence of internal and environmental filtering on these communities. It appears that environmental filters are important in both high‐elevation sites in Peru and in savanna sites in Brazil. Internal filters are present in Brazilian forest, but also present throughout most of the Peruvian elevation gradient, and not just at the low‐elevation sites. Our results lend initial support to previous findings that communities are not randomly assembled (Kraft et al. 2008; HilleRisLambers et al. 2012). While we observed patterns in filtering using a single trait, further investigation of community assembly across these gradients may benefit from using multiple traits (Kluge and Kessler 2011; Hoiss et al. 2012) and associating *T*‐statistics with other analytical tools such as generalized dissimilarity modeling (Ferrier et al. 2007; Graham and Fine 2008). In the future, expanding the analyses to also include rare and less dominant species will help identify the roles of niche‐based processes (Baraloto et al., 2012) and phylogenetic partitioning (Asner et al. 2014b) in influencing the biodiversity within and across in megadiverse species assemblages in the tropics. Below, we discuss specific patterns in filtering observed across the two contrasting gradients.

#### Internal filtering

In Brazil, *T_*ip.ic was lower in the forest and forest‐transition plots than in the two savanna plots. Individuals belonging to forest and forest‐like plots had more similar trait values than individuals drawn randomly from the community, which could indicate niche packing. Indeed, in examining functional trait space and the latitudinal diversity gradient, Lamanna et al. (2014) found that the overall temperate trait hypervolume was larger than the overall tropical hypervolume, indicating that niche packing is strong in tropical forests. Through examining functional diversity metrics related to inter‐ and intraspecific variation in the Brazilian pampa forest community, Carlucci et al. (2015) found that conspecific individuals varied more in communities where trait variability was typically high, that is, in the large forest patch, and less where trait variability was typically low, that is, in the small forest patches. Additionally, in Brazilian savannas, a phenological study showed that grasses are under competition pressure that can result in larger niche segregation, allowing coexistence between them (Ramos et al. 2014).

Niche differences (reflected in species trait values) can arise from differences among species in their effect on and response to biotic limiting factors like consumers and mutualists (HilleRisLambers et al. 2012). Leaf area index was much higher (and thus less light was available) in the forest plot than in the savanna plots (Table 1). Thus, our observation of internal filtering in forests compared to savannas could relate to increased light competition in the forest, perhaps even affecting species selection among saplings (Spasojevic et al. 2014). Indeed, light availability was shown to be an important determinant of species coexistence in closed moist forests, where this factor is limiting and likely to trigger competition (Hubbell et al. 1999; Poorter et al. 2006).

In Peru, internal filtering was observed across 80% of the plots for sun leaves: *T_*ip.ic was significantly lower than the null model, indicating less intraspecific variation than expected from the null model. This might be related to high species turnover along the gradient, or competition. A high level of species competition within these plots is expected as they are within old‐growth forests with limited light availability (Hubbell et al. 1999; Poorter et al. 2006). An alternative explanation to competition as the main internal filtering process is that community‐scale internal filtering is nonrandomly affected by site‐specific species (Baldeck et al. 2013; Asner et al. 2014b; Asner and Martin 2016).

#### External filtering

In Brazil, we found very high individual levels of variation leading to a high *T_*ic.ir in the savanna plot. This could be due to a very low environmental filtering. However, this site was also the only one without significant differences between *T_*ip.ic and null models. High individual variation at both the species and community levels is difficult to interpret in terms of community assembly.

In Peru, *T_*ic.ir was lower than expected in five plots: two plots bracketing the cloud immersion zone and two plots in lower‐montane and lowland rainforest. As a significant difference in *T*_ic.ir compared to the null model indicates low total variation of LMA in the community but high intraspecific variation along the gradient, species in these five Peruvian plots might be limited by environmental filters. Environmental filtering can shape the functional composition of highly diverse tropical forests (Fortunel et al. 2014) and may be particularly strong in local communities differing in topographic position (Kraft et al. 2008) or across strong edaphic or climatic gradients (Cavender‐Bares et al. 2006; Fine and Kembel 2011). In Peru, the distinct seasonal and diurnal cycles of cloud immersion at the Andes/Amazon transition (Halladay et al. 2012) potentially affect environmental filtering at the extremes of the cloud forest zones. While Ledo et al. (2013) found that both topographic conditions and forest structure contribute to small‐scale microhabitat partitioning of woody plant species in a Peruvian tropical cloud forest, canopy species were most correlated with the distribution of environmental variables in the cloud forest. Moreover, the strongest environmental controls on simulated gross primary productivity in the Peruvian cloud forest were variation of photosynthetic active radiation and air temperature, likely varying as a result of elevation and the local prevalence of cloud cover (van de Weg et al. 2014).

At the lower elevations in Peru, we suggest that low individual variation was driven by variation in soil fertility, which is strongly controlled in these sites by geologic substrate (Asner and Martin 2011; Asner et al.*,* 2015). Indeed, along this gradient in particular, total soil nitrogen and plant‐available nitrogen increase with elevation (Nottingham et al. 2015) and P limitation drives N:P variations among lowland Amazonian plots, explaining in particular variations between TAM‐05 and TAM‐06 (Asner and Martin 2011; Fisher et al. 2013).

Importantly, similar patterns of external filtering were not observed at the species level (*T_*pc.pr), when only interspecific variation was used. This supports the importance of incorporating intraspecific variation in community studies (Jung et al. 2010; Albert et al. 2011; Bolnick 2011). In further support, intraspecific variation did account for a significant part of the total trait variance as shown through variance partitioning (Fig. S3). This result is consistent with a growing body of literature advocating the use of both individual and species‐specific traits to investigate community assembly mechanisms (Jung et al. 2014; Albert 2015; Enquist et al. 2015).

## Conclusions

Our results confirm LMA patterns described in the literature, such as higher LMA in drier, and more UV‐exposed environments. Interpreted in light of the leaf economic spectrum, these variations indicate faster‐end strategies in nutrient‐rich environments where competition is assumed strong, and slower‐end strategies in less productive, climatically and substrate‐constrained environments. Importantly, we found that intraspecific variation accounted for a significant part of the total variation in LMA and that differences among species and genera were more important in determining trait patterns in Peru compared to Brazil. As a result of variation in LMA across gradients, we were able to observe the presence of both internal and environmental filtering in these communities. These results assume that a large amount of variation in plant distribution and performance is explainable due to inter‐ and intraspecific variation in LMA. We recognize, however, that LMA is only one trait in the LES, and only one of many important functional traits, some of which are only loosely correlated with LMA. Thus, we hope that our study will motivate future work using multifunctional trait analyses via inter‐ and intratrait variance decomposition to understand mechanisms driving functional diversity within the tropics in light of climate change (Duque et al. 2015; Rehm and Feeley 2015) and to inform biodiversity conservation and sustainable use policies.

## Conflict of Interest

None declared.

## Data Accessibility

R scripts are included as online supporting information. Data are to be available via the TRY database.

## Supporting information


**Appendix S1.** R code used for analyses.

**Figure S1.** Leaf mass per area (LMA) values along the environmental gradients.
**Figure S2.** The Sorensen index of dissimilarity calculated for each pair of plots and regressed against the difference in light area index along the vegetation gradient in Brazil and difference in elevation along the gradient in Peru.
**Figure S3.** Comparison of variance partitioning across phylogenetic levels along both gradients.
**Table S1.** Description of the different null models used to calculated significance for *T*‐statistics.
**Table S2.** The percentage of trees belonging to each category of deciduousness within each plot along the Brazilian gradient.
**Table S3.** Individual variance components used in the calculation of *T*‐statistics.
