# Supplementary material for: Examining variation in the leaf mass per area of dominant species across two contrasting tropical gradients in light of community assembly
Source: Ecol Evol. 2016 Jul 22;6(16):5674–89. doi: 10.1002/ece3.2281 (PMC4983583; doi:10.1002/ece3.2281)
Supplement: Supplementary file 2 — Figure S1. Leaf mass per area (LMA) values along the environmental gradients. Figure S2. The Sorensen index of dissimilarity calculated for each pair of plots and regressed against the difference in light area index along the vegetation gradient in Brazil and difference in elevation along the gradient in Peru. Figure S3. Comparison of variance partitioning across phylogenetic levels along both gradients. Table S1. Description of the different null models used to calculated significance for T‐statistics. Table S2. The percentage of trees belonging to each category of deciduousness within each plot along the Brazilian gradient. Table S3. Individual variance components used in the calculation of T‐statistics. [file ECE3-6-5674-s002.docx]

## Supporting Information

Article title: **Examining variation in the leaf mass per area of dominant species across two contrasting tropical gradients in light of community assembly**

Authors: Margot Neyret, Lisa Patrick Bentley, Imma Oliveras, Beatriz S. Marimon, Ben Hur Marimon-Junior, Edmar Almeida de Oliveira, Fábio Barbosa Passos, Rosa Castro Ccoscco, Josias dos Santos, Simone Matias Reis, Paulo S. Morandi, Gloria Rayme Paucar, Arturo Robles Cáceres, Yolvi Valdez Tejeira, Yovana Yllanes Choque, Norma Salinas, Alexander Shenkin, Gregory P. Asner, Sandra Díaz, Brian J. Enquist and Yadvinder Malhi

Fig S1: Leaf mass per area (LMA) variation along the environmental gradients in (a) Brazil and in Peru for (b) sun and (c) shade leaves.

Each dot represents the mean LMA for one tree.


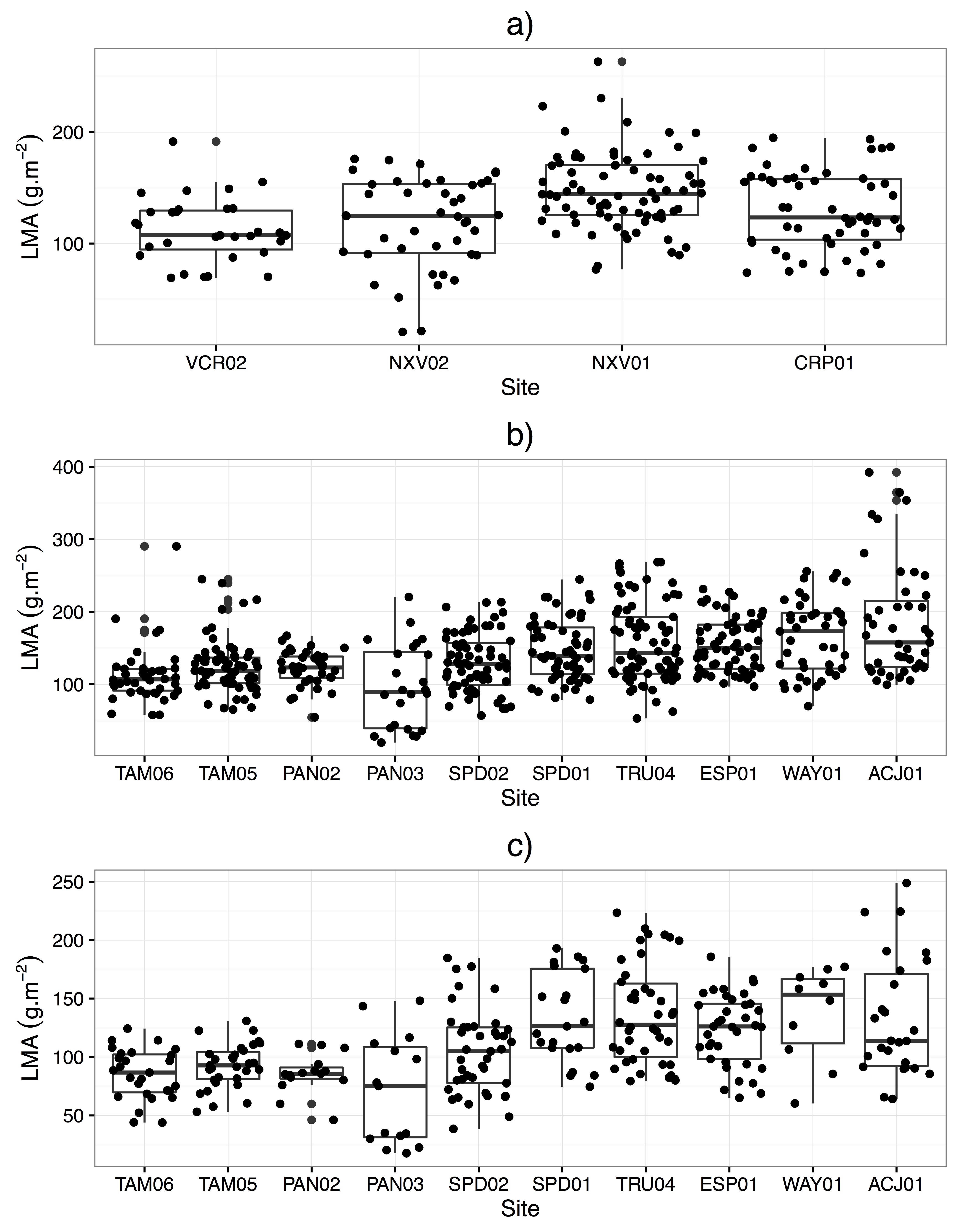


Fig. S2: To determine species turnover along the gradients, the Sorensen index of dissimilarity was calculated for each pair of plots and regressed against the difference in (a) light area index (LAI; Table S2) along the vegetation gradient in Brazil and (b) elevation (m) along the Peruvian gradient. Statistics (*p*-value and R^2^) were obtained from a Mantel test. In the vegetation gradient, the lowest dissimilarity was 33% between NXV-01 and CRP-01 and the forest plot (VCR-02) had a dissimilarity of 88% with its most similar plot, NXV-02. b) In the elevation gradient, the turnover was very high, with the lowest dissimilarity of 63% between two neighboring plots (ESP-01 and WAY-01) and many plots sharing no species (dissimilarity of 100%).


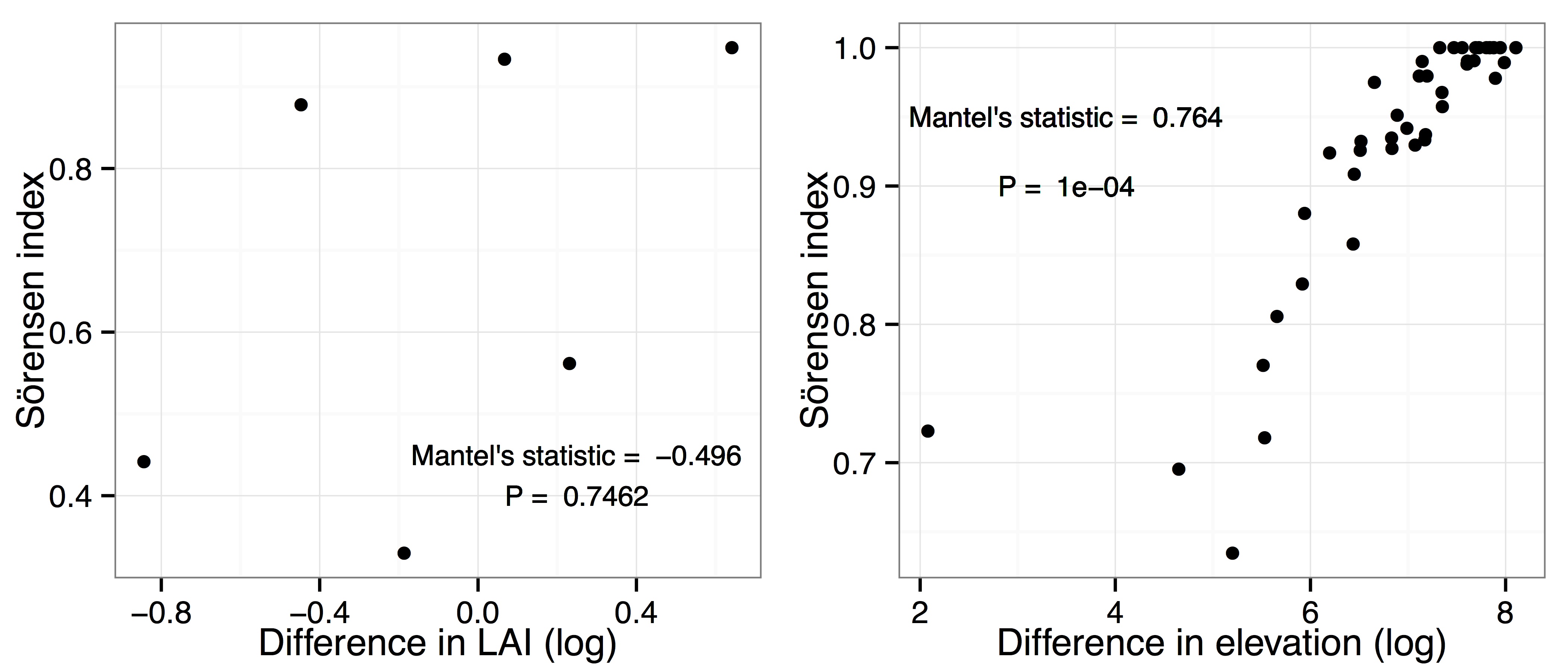


Fig. S3: Comparison of variance partitioning across phylogenetic levels along both gradients. The variance partitioning was calculated using the *lme* and *varcomp* functions in R (packages *ape* and *nlme*). Intra-specific variation (individual + within individual) accounts for more than 25% of the total variation along both gradients.


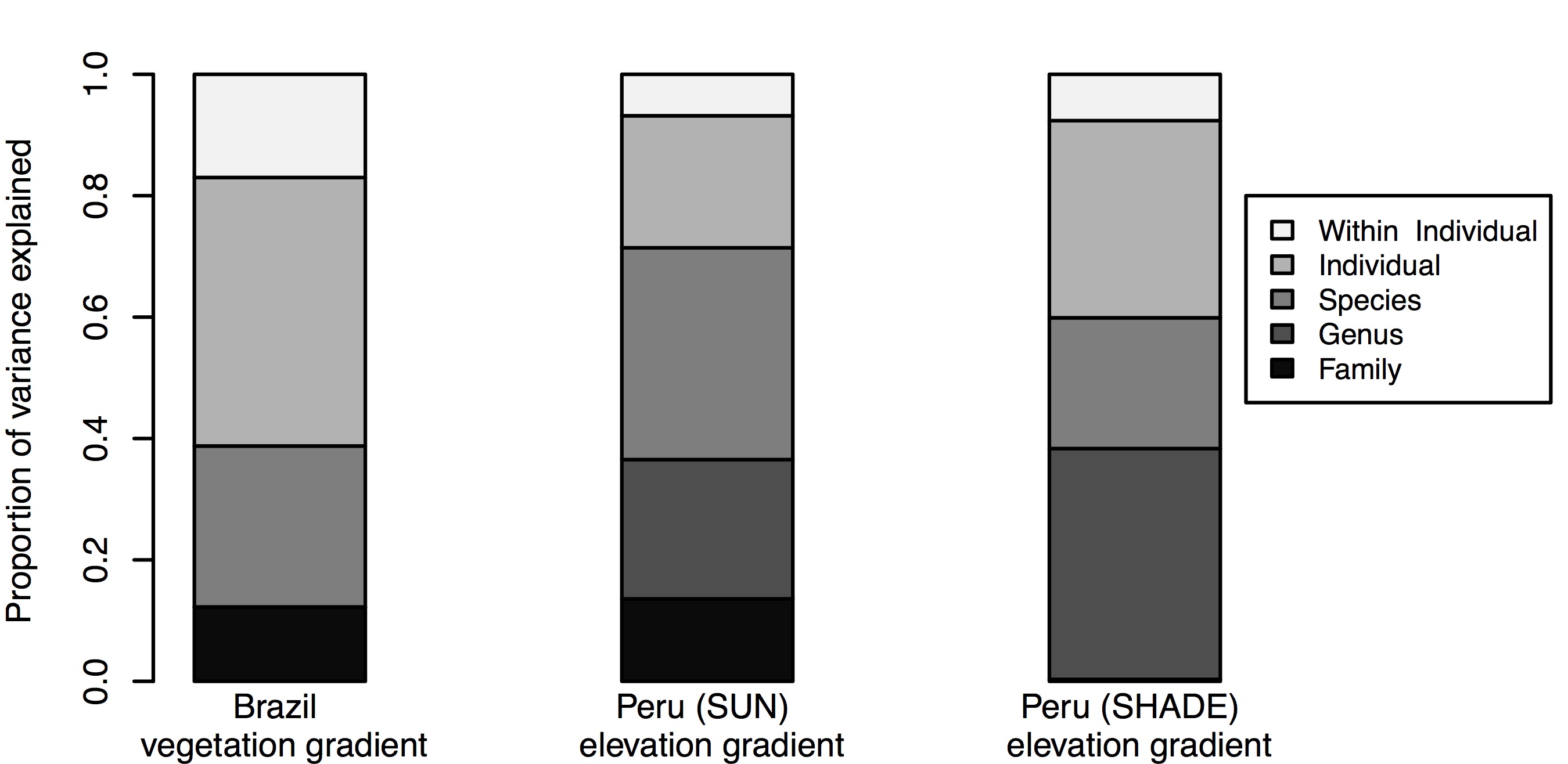


Table S1: Description of the different null models used to calculate significance for T-statistics (adapted from Taudière & Violle 2015).

| **T-statistic** | **Null hypothesis** | **Randomisation** |
| --- | --- | --- |
| *T_ip.ic* | Trait value distribution is independent from species identity: there is no internal filtering. | Individual trait values are shuffled within each community. |
| *T_ic.ir* | Individual trait value  distribution is drawn randomly from the regional pool: there is no external filtering acting on individuals. | Individual trait values are shuffled within the regional pool, keeping the number of individuals in each community constant. |
| *T_pc.pr* | Species mean trait value distribution is drawn randomly from the regional pool: there is no external filtering acting on species. | Each individual is assigned the mean value of the species; the values are then shuffled within the regional pool, keeping the number of individuals in each community constant. |

Table S2: The percentage of trees belonging to each category of decicuousness within each plot along the Brazilian gradient. To determine deciduousness, trees belonging to a total of 65 species were counted in each plot (260 trees were included). Their deciduousness was determined from the literature (Pirani *et al*., 2009 ; Divino, 2010 ; Teixeira and Oliveira-Filho, 2010 ; Campos, 2007 ; Silva, 2012). Trees in CRP-01 appear to be mostly deciduous, or semi-deciduous; trees were mostly semi-deciduous in NXV-01 and mostly evergreen in NXV-02 and VCR-02. This distribution was significantly different from a random distribution (chi-square test, p < 2.10^-6^). Leaf area index is also included for each site.

|  | CRP-01  Dry savanna | NXV-01  Savanna | NXV-02  Transition | VCR-02  Forest |
| --- | --- | --- | --- | --- |
| % Evergreen (continuous or seasonal) | 12.3 | 27.1 | 58.2 | 47.2 |
| % Semi-deciduous | 37.0 | 40.0 | 23.6 | 36.1 |
| % Deciduous | 50.6 | 33.3 | 18.2 | 16.7 |
| Leaf area index | 1.51 | 2.34 | 2.77 | 3.41 |

Table S3 : Individual variance components as used in the calculation of T-statistics. Variances are calculated for log-transformed LMA.

**σ^2^_ip_mean_**: mean individual variation for species in the community (i.e. in the plot)

**σ^2^_pc_**: inter-specific variation in each plot

**σ^2^_cr_** : inter-community variation in the region

**σ^2^_ic_** : individual variation in the community

**σ^2^_pr_** : inter-specific variation in the region

**σ^2^_ir_** : individual variation in the region

**T_ip.ic =_ σ^2^_ip_mean/_ σ^2^_ic ;_ T_ic.ir =_ σ^2^_ic_ / σ^2^_ir ;_ T_pc.pr =_ σ^2^_pc_ / σ^2^_pr_**

|  | **Site name** | **σ^2^_ip_mean_** | **σ^2^_pc_** | **σ^2^_cr_** | **σ^2^_ic_** | **σ^2^_pr_** | **σ^2^_ir_** | **T_ip.ic_** | **T_ic.ir_** | **T_pc.pr_** |
| --- | --- | --- | --- | --- | --- | --- | --- | --- | --- | --- |
| **Vegetation gradient** | CRP-01 | 3,01.10^-2^ | 5,75.10^-2^ | 1,69.10^-2^ | 6,69.10^-2^ | 7,04.10^-2^ | 1,14.10^-1^ | 4,49.10^-1^ | 5,88.10^-1^ | 8,16.10^-1^ |
|  | NXV-01 | 2,15.10^-1^ | 4,71.10^-2^ |  | 2,34.10^-1^ |  |  | 9,20.10^-1^ | 2,06 | 6,69.10^-1^ |
|  | NXV-02 | 1,92.10^-2^ | 8,99.10^-2^ |  | 5,64.10^-2^ |  |  | 3,40.10^-1^ | 4,96.10^-1^ | 1,28 |
|  | VCR-02 | 2,24.10^-2^ | 4,19.10^-2^ |  | 7,69.10^-2^ |  |  | 2,92.10^-1^ | 6,76.10^-1^ | 5,95.10^-1^ |
| **Elevation gradient (Sun)** | ACJ-01 | 3,56.10^-2^ | 1,40.10^-1^ | 5,21.10^-2^ | 9,01.10^-2^ | 1,09.10^-1^ | 1,43.10^-1^ | 3,95.10^-1^ | 6,32.10^-1^ | 1,29 |
|  | ESP-01 | 3,39.10^-2^ | 4,88.10^-2^ |  | 7,91.10^-2^ |  |  | 4,28.10^-1^ | 5,55.10^-1^ | 4,48.10^-1^ |
|  | PAN-02 | 1,79.10^-2^ | 4,14.10^-2^ |  | 5,12.10^-2^ |  |  | 3,49.10^-1^ | 3,59.10^-1^ | 3,80.10^-1^ |
|  | PAN-03 | 5,35.10^-1^ | 1,50.10^-1^ |  | 5,09.10^-1^ |  |  | 1,05 | 3,57 | 1,38 |
|  | SPD-01 | 2,62.10^-2^ | 6,82.10^-2^ |  | 1,02.10^-1^ |  |  | 2,58.10^-1^ | 7,13.10^-1^ | 6,26.10^-1^ |
|  | SPD-02 | 1,96.10^-2^ | 8,84.10^-2^ |  | 7,81.10^-2^ |  |  | 2,51.10^-1^ | 5,48.10^-1^ | 8,12.10^-1^ |
|  | TAM-05 | 1,52.10^-2^ | 5,85.10^-2^ |  | 1,27.10^-1^ |  |  | 1,20.10^-1^ | 8,92.10^-1^ | 5,37.10^-1^ |
|  | TAM-06 | 1,66.10^-2^ | 6,92.10^-2^ |  | 5,75.10^-2^ |  |  | 2,88.10^-1^ | 4,03.10^-1^ | 6,35.10^-1^ |
|  | TRU-04 | 2,62.10^-2^ | 1,18.10^-1^ |  | 1,12.10^-1^ |  |  | 2,34.10^-1^ | 7,84.10^-1^ | 1,08 |
|  | WAY-01 | 1,65.10^-2^ | 9,29.10^-2^ |  | 1,45.10^-1^ |  |  | 1,13.10^-1^ | 1,02 | 8,53.10^-1^ |
| **Elevation gradient (Shade)** | ACJ-01 | 2,47.10^-2^ | 1,22.10^-1^ | 7,36.10^-2^ | 7,79.10^-2^ | 1,30.10^-1^ | 1,68.10^-1^ | 3,17.10^-1^ | 4,64.10^-1^ | 9,37.10^-1^ |
|  | ESP-01 | 2,23.10^-2^ | 6,48.10^-2^ |  | 4,82.10^-2^ |  |  | 4,62.10^-1^ | 2,87.10^-1^ | 4,98.10^-1^ |
|  | PAN-02 | 2,17.10^-2^ | 4,17.10^-2^ |  | 5,13.10^-2^ |  |  | 4,24.10^-1^ | 3,05.10^-1^ | 3,20.10^-1^ |
|  | PAN-03 | 5,30.10^-1^ | 2,32.10^-1^ |  | 5,78.10^-1^ |  |  | 9,18.10^-1^ | 3,44 | 1,78 |
|  | SPD-01 | 2,57.10^-2^ | 5,96.10^-2^ |  | 1,30.10^-1^ |  |  | 1,97.10^-1^ | 7,76.10^-1^ | 4,58.10^-1^ |
|  | SPD-02 | 4,97.10^-2^ | 1,20.10^-1^ |  | 8,84.10^-2^ |  |  | 5,62.10^-1^ | 5,27.10^-1^ | 9,19.10^-1^ |
|  | TAM-05 | 2,02.10^-2^ | 3,65.10^-2^ |  | 9,84.10^-2^ |  |  | 2,05.10^-1^ | 5,86.10^-1^ | 2,80.10^-1^ |
|  | TAM-06 | 2,53.10^-2^ | 6,59.10^-2^ |  | 7,27.10^-2^ |  |  | 3,49.10^-1^ | 4,33.10^-1^ | 5,06.10^-1^ |
|  | TRU-04 | 3,88.10^-2^ | 8,85.10^-2^ |  | 1,30.10^-1^ |  |  | 2,99.10^-1^ | 7,71.10^-1^ | 6,80.10^-1^ |
|  | WAY-01 | 3,81.10^-2^ | 1,45.10^-1^ |  | 1,39.10^-1^ |  |  | 2,74.10^-1^ | 8,28.10^-1^ | 1,12 |

**References:**

**De Campos ÉP**. **2007**. Fenologia e chuva de sementes em Floresta Estacional Semidecidual no município de Viçosa, Minas Gerais, Brasil.

**Dias HCT., de Oliveira-Filho A**. **1996**. Fenologia de quatro espécies arbóreas de uma Floresta Estacional Semidecídua Montana em Lavras. MG. *Cerne 2.1*: 66–88.

**Pirani FR., Sanchez M., Pedroni F**. **2009**. Phenology of a tree community in a cerrado sensu stricto., Barra do Garças., Mato Grosso State. Brasil. *Acta Botanica Brasilica* **23**: 1096–1110.

**Silva J**. **2012**. Árvores do Cerrado: sentido restrito. Guia de campo. *Brasília: Rede de Sementes do Cerrado*.

**Silverio D**. **2010**. Efeito do fogo e do substrato sobre a fenologia de espécies lenhosas em duas fitofisionomias de Cerrado no Parque Municipal do Bacaba, Nova Xavantina, Mato Grosso, Brasil.
